# Application of antibodies to recombinant heat shock protein 70 in immunohistochemical diagnosis of *mycobacterium avium subspecies paratuberculosis* in tissues of naturally infected cattle

**DOI:** 10.1186/s13620-017-0088-7

**Published:** 2017-03-24

**Authors:** Julius Boniface Okuni, David Patrick Kateete, Moses Okee, Anna Nanteza, Moses Joloba, Lonzy Ojok

**Affiliations:** 1 College of Veterinary Medicine, Animal Resources and Biosecurity, Makerere University, P.O. Box 7062, Kampala, Uganda; 2 College of Health Sciences, Makerere University, P.O. Box 7072, Kampala, Uganda

**Keywords:** Johne’s disease, *Mycobacterium avium subspecies paratuberculosis*, HSP70, Immunohistochemistry

## Abstract

**Background:**

Detection of *Mycobacterium avium subspecies paratuberculosis* (MAP) infection is key to the control of Johne’s disease. Immunohistochemistry is one of the methods of detection of MAP infection in tissues. However, unavailability of commercial antibodies that can detect the organism is a limiting factor for the use of immunohistochemistry. This study was aimed at developing an immunohistochemistry method to diagnose MAP in infected tissues using antibodies against MAP recombinant heat shock protein 70kd.

**Results:**

MAP Heat shock protein 70 gene was amplified and cloned into an expression vector, Champion pET-SUMO, then expressed in *E coli*, purified and used to produce polyclonal rabbit antibodies against the Heat shock protein. Immunohistochemistry was performed in 35 MAP infected tissues with anti-HSP70 polyclonal antibodies. All 35 MAP infected tissues were positive for MAP within macrophages, epithelioid cells and giant cells either in clumps or singly as individual bacilli. No positive staining was seen in the three uninfected normal tissues and in MAP infected tissues where primary antibodies were substituted with PBS or pre-immune serum from the same rabbit.

**Conclusion:**

Anti-HSP70 produced in this study offers an opportunity for improved diagnosis, screening of MAP in animal tissues and in studies on the pathogenesis of MAP

**Electronic supplementary material:**

The online version of this article (doi:10.1186/s13620-017-0088-7) contains supplementary material, which is available to authorized users.

## Background

*Mycobacterium avium* subspecies *paratuberculosis* (MAP) is the cause of Paratuberculosis or Johne’s disease in cattle, buffaloes, sheep, goats, deer and other species [1]. MAP is regarded as a possible zoonotic pathogen since there are incriminating reports on its involvement in Crohn’s disease [2, 3]. Diagnosis of paratuberculosis or MAP infection is key to the control of Johne’s disease [4, 5]. Confirmatory diagnosis is based on culture, antigen detection systems and nucleic acid based amplifications to support gross and microscopic lesions in suspected cases [6]. In low prevalence areas, these specialised techniques might not be in place, so diagnosis of the infection may be done on post-mortem and histological examinations [7]. Unfortunately, in some cases of MAP infection, the gross and microscopic lesions might not be well developed and need the support of other techniques such as Ziehl Neelsen acidfast staining (ZN), PCR, in situ hybridisation and immunohistochemistry (IHC) [8]. ZN staining is cheap and easier to perform but its sensitivity and specificity is low in comparison with the other three [9]. PCR and in situ hybridisation need special equipment which are lacking in many laboratories; whereas IHC, though technically simpler, is limited by the unavailability of specific commercial antibodies against the pathogen. Thus there is continuing search for new antigens and antibodies for use in IHC detection of MAP [10]. Recombinant DNA technology offers an opportunity to produce large quantities of antigens which are difficult to purify in sufficient quantities in their wild forms [11]. Therefore, finding new antigens and use of recombinant DNA technology are the best hope for immunodiagnostic of paratuberculosis as it is for many other diseases.

Heat shock proteins are antigenic proteins that can initiate immune responses, deliver antigens into major histocompatibility complex I (MHCI) pathways, elicit pro-inflammatory responses in antigen presenting cells and facilitate folding and unfolding of cytosolic proteins [12, 13]. They are produced in different molecular weights by almost all kinds of cells in response to cell stress but each of them is different from the other [14]; hence they can be considered as markers of the cells or organisms that produce them [14]. Small heat shock proteins are therapeutic because of their chaperone activity [15]. Heat shock protein 70 (HSP70) of mycobacteria are antigenic, stimulating immune response against mycobacteria [16]. One study [17] showed that MAP HSP70 could be an effective subunit vaccine against MAP. This is via the stimulation of dendritic cells and a strong T-helper 1 cell response [18, 19]. HSP70 fusion protein is particularly strongly antigenic [18]. HSP70 and other chaperonin genes are also potential drug targets [20]. Differential expression of HSP65 and HSP70 during different stages of paratuberculosis has been reported [21], suggesting that they might act as markers of different stages of infection and the type of disease [22]. When epitope specificities of Ig G antibodies were compared to HSP60/65 in healthy individuals and patients with Chronic heart disease and inflammatory disease [23], it was found that the epitopes varied specifically in different diseases at different stages, providing more evidence that HSP70 antibodies can be markers of disease progression.

Many studies have attempted to determine the potential use of heat shock proteins for immunodiagnosis with various successes. For instance, one of the molecules from HSP70 family for *Typanosoma cruzi* was able to distinguish between infected and non-infected persons and between treated and non-treated persons [24]. That finding is supported by another study [25]. Although the use of HSP70 from MAP in serological diagnosis has been explored [10], there is hardly any report on IHC using antibodies against MAP HSP70 in naturally infected cattle.

The purpose of this study was to evaluate antibodies against recombinant MAP HSP70 expressed in Champion PET-SUMO expression system for IHC diagnosis of MAP in infected tissues.

## Method

The gene for heat shock protein 70 was amplified as follows: Primers ehsp70f (5′-GGG GTA CCC CCT ATG GCT CGT GCG GTC GGT ATC-3′) and ehsp70r (5′-CCC AAG CTT GGG TCA CTT GGA CTC CCG GTC ATC G-3′) were designed in frame with the initiation codon of SUMO protein in Champion PET-SUMO vector (Invitrogen, Life technology). HSP70 gene was amplified from DNA extracted from a Ugandan isolate of MAP. The set up included 50μl PCR reaction containing 5μl of custom master mix, 5μl of forward and reverse primers each, 35μl of PCR water, 0.5μl of Taq polymerase and 2μl of DNA template. The thermal profiles of the reaction consisted of initial denaturation at 94°C for 5 min, 35 cycles of denaturation at 94°C for 30 s, 63°C for 60 s, 72°C for 120 s, and a final extension of 72°C for 10 min. The reaction was cooled to 4°C and then removed. The success of the PCR reaction was analysed using 1% agarose electrophoresis. The PCR product was then ligated into the expression vector Champion PET-SUMO according to the manufacturer’s instructions. The ligation product was transformed into Mach1 T1 cells. Successful transformation was confirmed by restriction endonuclease digestion using *AflII*(New England biolabs) and PCR. After confirmation, the recombinant plasmid in the correct orientation was transformed into BL21 D3 cells. The plasmid containing SUMO+ CAT (chlorophenical acetyl transaminase) was used as a control for the expression. Expression of the protein was done according to the manufacturer’s instruction and was confirmed on SDS-PAGE stained with Coomasie blue and Western blot then probed with anti-His antibodies. HSP70 was purified under native conditions as a fusion to SUMO protein using Pro-Bond Ni-NTA agarose protein purification system (Invitrogen, Life technologies incorporated) according to the manufacturer’s instruction manual. The SUMO fusion tag was removed from the HSP70 using SUMO protease digestion. Fractions of the digested protein were pooled together and concentrated using amicon® ultra centrifugal filter columns of 50 Kd. The purity of the protein was checked through SDS PAGE analysis. The protein was then constituted with a storage buffer containing 50mM Tris-HCl (pH 7.9), 0.1mM EDTA, 0.1mM DTT, 50% glycerol and 0.1M NaCl (TGED +0.1M NaCl). Five hundred millilitres of the solution containing 350μg of HSP70 was emulsified with an equal volume of Aluminium hydroxide adjuvant (alhydrogel®, Brenntag Biosector, Fredriksund, Denmark). Two New Zealand white rabbits were each injected with 1ml of the emulsified HSP70 at about 4 -5 sites over the suprascapular region. Another rabbit was used as control and immunised with sterile phosphate buffered saline emulsified with the adjuvant alone, while two others were immunised with whole cell suspensions of killed MAP isolates. Boosting of immune response was done with 150 μg of the protein on 14th and 28th days after initial vaccination. The control rabbit received the same treatment as before (PBS and adjuvant, cell suspension plus adjuvant) each time during the boosting. The rabbits were bled on the 10th and 24th days after immunisation and terminal bleeding was done on the 42nd day of the experiment. To confirm the production of polyclonal antibodies, western blot was performed to probe the antigens.

For Immunohistochemical staining of MAP using antibodies against HSP70 and whole cell suspensions; 5 micrometre thick sections were cut on Poly L Lysine coated slides and IHC was carried out as described by Ramos-Vara and Miller [26] with a few modifications. Antigen retrieval was carried out using citrate buffer treatment at pH 6.2. For the antigen retrieval, Di-Sodium citrate buffer (pH 6.2) was preheated for 10 min in a microwave oven set at 1000W; the microwave oven was adjusted to 700W then a rack containing the slides was immersed into the buffer and heated for 20 min. The beaker containing the buffer plus the slides were removed and allowed to cool for 20 min. The slides were then washed three times. 100μl of normal goat serum was used for blocking for 30 min at room temperature (RT) followed by the addition of 100μl of antiHSP70 antibodies (1/1000) and antibodies against whole cell suspensions (1/250). The sections were incubated at 4°C overnight. Pre-immune rabbit serum was used as a negative control instead of the primary antibodies (1/1000). Secondary goat anti-rabbit antibodies coupled to horseradish peroxidase was used at a dilution of 1/800 and sections were incubated at RT for one hour. DAB (3, 3, diaminobenzidine) solution was prepared from Sigma FastTM DAB and urea tablets as described in the material data sheet. DAB was applied on the tissue for 2-3 min.

Thirty-five histologically positive cases of JD and two non-MAP tuberculous cases comprising a case of avian TB and a bovine TB were stained with antibodies against HSP70. Three cases that were without any granulomatous lesion were also included as controls. Twenty five of the 35 cases were obtained during a Paratuberculosis survey in Kampala abattoirs and were found to have characteristic microscopic lesions as well as staining positive for Ziehl Niesen stain (See the Additional file 1: Table S1). Additionally, one case was from a case of Johne disease confirmed by necropsy, culture and PCR. The remaining nine cases included intestinal tissue blocks of 4 cattle and 5 sheep from Germany which was a kind gift from Prof. Dr. Manfred Reinacher of the Insitute for Veterinary Pathology, University of Giessen, Germany (see Additional file 1: Table S1).

## Results

**Fig. 1:**
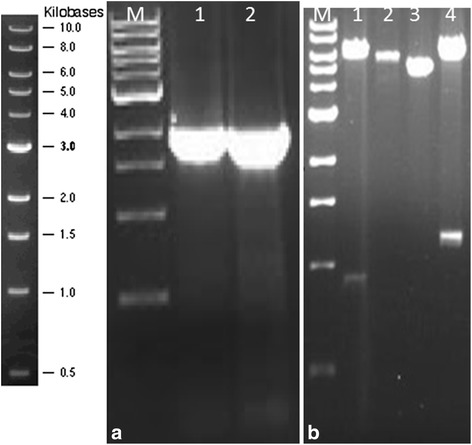
An agarose gel electrophoresis of HSP70 gene amplified product and restriction analysis of PET-SUMO + HSP70 recombinant plasmid. **a** The open reading frame of HSP70 gene, 1872bp in length was amplified. The PCR product in **a** was later cloned in PET-SUMO vector (Invitrogen). **b** The restriction patterns of HSP70 cloned in frame with PET-SUMO vector. From left, lane 1 is the DNA ladder, lanes 2 is a recombinant with the gene in a 3’-5’direction (wrong orientation) showing two bands 6720bpand 910bp, lane 3 and 4 are non-recombinant plasmids without the insert(≈5643bp), lane 5 is the plasmid with an insert in the correct 5’-3’ orientationshowing two fragments 6445bp and 1185bp. The DNA ladder used is the NEB 1kb ladder. The bar chart to the left of A shows the sizes of the DNA fragments on the ladder, which in turn shows the size of the amplified products in gel **a** and **b**

**Fig. 2:**
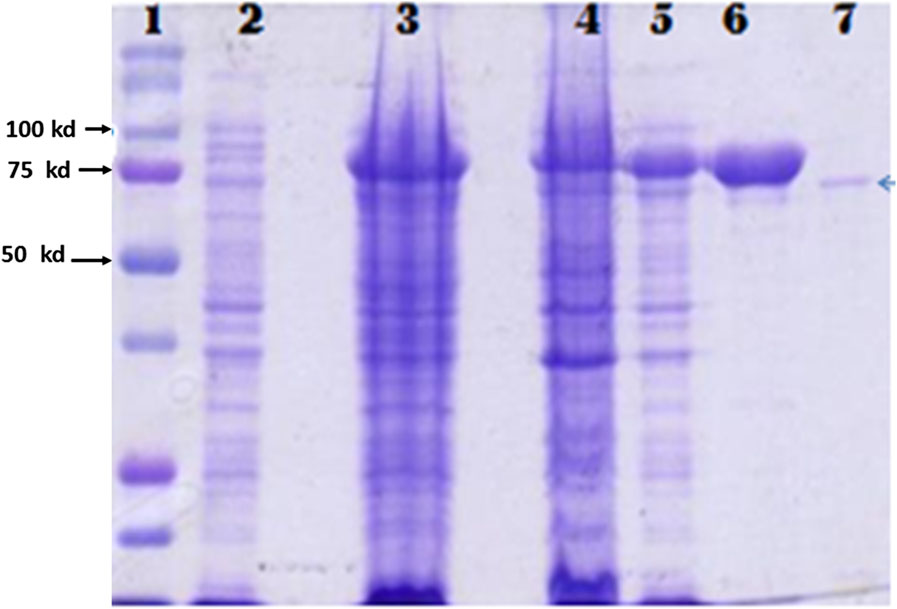
An SDS-PAGE gel analysis showing the expression of HSP70 before and four hours post induction plus the purified fusion protein. Lane 1 is a protein standard, lane 2 is pre-induction whole cell lysate, lane 3 is a whole cell lysate after 4 h post induction, lane 4 is the pellet after the cells were lysed under native conditions, lane 5 is the supernatant under native conditions, lane 6 is the purified HSP70 with the SUMO protein tag while lane 7 shows HSP70 after the SUMO fusion tag was removed with SUMO protease. The fusion protein was 83 Kd before the SUMO tag was removed (lane 6) by a sumo protease, resulting in a pure HSP70 recombinant protein of 70 Kd (lane 7)

**Fig. 3:**
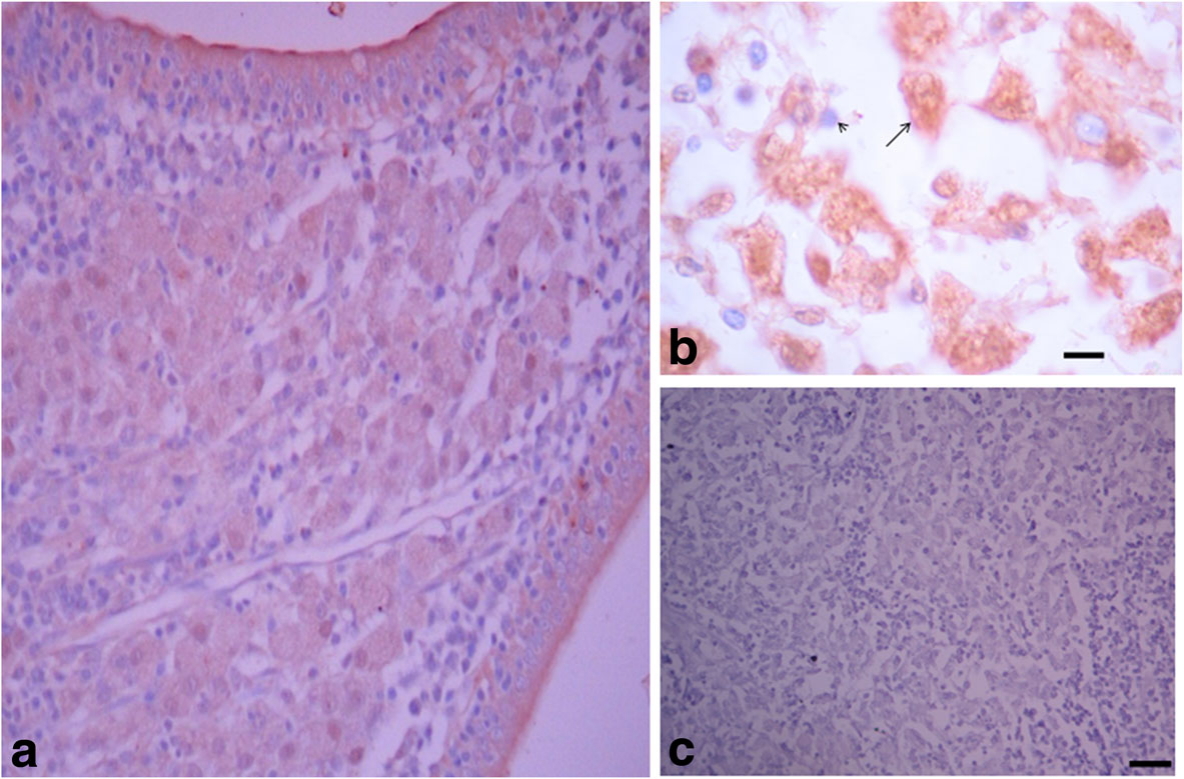
Immunohistochemical staining of *Mycobacterium avium subsp. paratuberculosis* in bovine intestinal mucosa using anti-MAP HSP70 antibodies. **a** A section through the mucosa showing many macrophages containing mycobacteria staining positive with Anti-HSP70 antibodies (DAB substrate-chromagen, and haematoxylin counter staining). **b** Higher magnification of **a**. Macrophages (arrow with long tail) and epithelioid cells, stained immunohistochemically using anti-HSP70 antibodies. Note the diffuse character of the staining within the cells. Bar = 17.5μM. **c** A negative control section in which the Primary antibodies were substituted by pre-immune rabbit serum. No positive staining was observed (DAB substrate-chromagen, and haematoxylin counter staining). Bar = 140μm

**Fig. 4:**
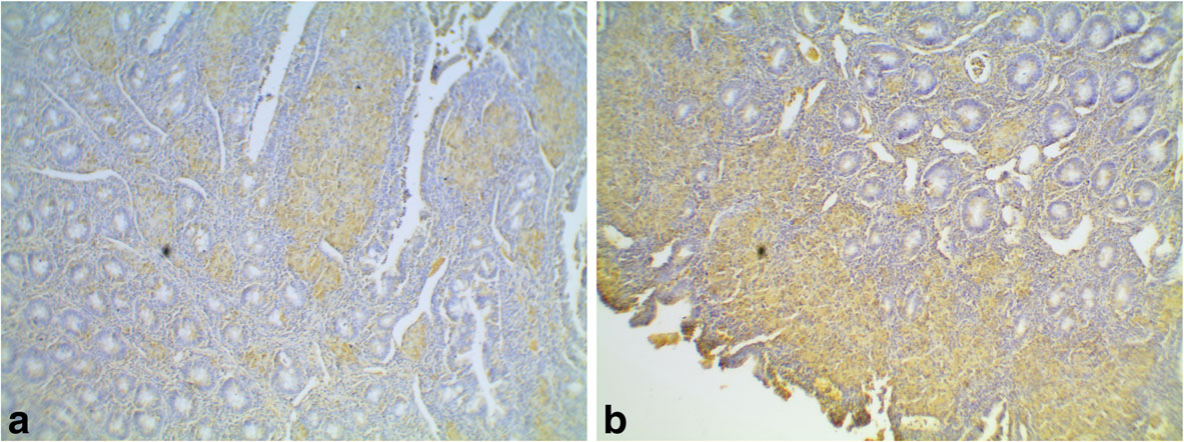
Immunohistochemical staining of Mycobacterium avium subspecies paratuberculosis in the mucosal tissue of bovine. The brown areas stained are areas containing large numbers of macrophages and epithelial cells containing the bacteria

**Fig 5:**
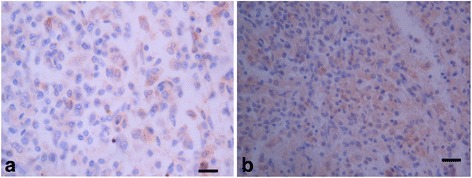
Positive immunohistochemical staining of bovine and avian tuberculous lesions with antibodies against recombinant MAP HSP70. **a** Bovine lymph node with bovine tuberculosis. **b** Chicken spleen with avian tuberculosis. Light brown staining shows macrophages and epithelioid cells containing tuberculous mycobacteria. Note that in both A and B, the staining is weaker than in the case of MAP infected macrophages. HSP70 antibodies, with DAB substrate-chromagen, and haematoxylin counter staining. Bar = 35μm

The amplification of HSP70 gene resulted into a PCR product of 1872bp PCR product (Fig. 1a). The restriction analysis of the PET-SUMO+ HSP70 recombinant plasmid showed two bands of 6445 base-pairs and 1185bp for the gene in a correct 5’-3’ orientation; and 6720 base-pairs and 910 base-pairs with the gene cloned in the opposite 3’-5’ orientation after digestion with *AflII* (Fig. 1b). When the recombinant gene was expressed, a band of 83kd was observed with increasing intensity on SDS-PAGE from 1-4 h post induction. Optimal expression was observed between 3 and 4 h post induction. This protein was cut into a 70kd protein (HSP70) and a 13kd protein (SUMO) using SUMO protease (Fig. 2). Immunohistochemical staining with anti-HSP70 polyclonal antibodies showed positive staining in all 35 MAP infected tissues. MAP was stained within macrophages, epithelioid cells and giant cells either in clumps or singly as individual bacilli. Figure 3 shows specific staining of infected cells in a heavily infected ileocaecal mucosal villus, specifically in the lamina propria. Most of the infected cells showed a diffuse staining of the cytoplasm, suggesting that large amounts of HSP70 were expressed by the bacteria (Figs. 3 and 4). Positive staining of acid-fast bacteria was also seen in two tuberculous lesions from avian and bovine TB (Fig. 5). The acid fast organisms in the avian and bovine tissues had not been typed and were assumed to be *M. avium* and *M. bovis* respectively. No positive staining was seen in the three apparently normal tissues and in MAP infected tissues where primary antibodies were substituted with PBS or pre-immune serum from the same rabbit. Positive staining of MAP was also seen with positive control antibodies raised by immunisation of rabbits with whole cell suspensions of MAP.

## Discussion

Positive staining of MAP was obtained in all infected tissues. Thus antibodies against MAP HSP70 are sensitive in the immunohistochemical diagnosis of MAP. The staining showed both individual bacilli, clumps of bacilli and diffuse staining within the cytoplasm [10] stated that HSP70 is a soluble antigen which easily diffuses out of the mycobacterium. This implies that HSP70 also diffuses outside the infected macrophages. Thus antibodies to HSP70 could be used for ELISA, IHC and electron microscopy [27]. Since there are no commercial antibodies against MAP available for use at the moment, despite the numerous studies including the ones cited in this paper, the need to test putative diagnostic antigens is crucial. Immunohistochemistry has been shown to be more sensitive and specific than ZN staining [28–30]. In this study HSP70 was expressed as a fusion protein with SUMO protein (HSP70 + SUMO) unlike in previous studies where it was produced as Histidine tagged fusion protein [10, 27]. The differences in the type and size of the fusion tag would be expected to result in different conformational structure, hence different epitopes. However polyclonal HSP70 antibodies have not resulted into specific MAP staining since positive staining was also observed with a bovine and avian tuberculous lesions though these had weaker signals than MAP infected tissues. This is not surprising considering the promiscuous character of most polyclonal antibodies [26]. Cross reactivity of other MAP antigens with other mycobacteria have been observed using polyclonal antibodies [28] but there is possibility that epitope mapping of this protein could unravel specific epitopes for MAP. Cross reactivity of both polyclonal and monoclonal antibodies has been reported for other MAP antigens except for HSP32 subunit which is so far able to distinguish between MAP and some related mycobacteria [29]. Despite the cross reactivity of the recombinant HSP70, this antigen still offers an opportunity for detection of MAP and other mycobacterial infection using immunohistochemistry in suspicious cases where a negative ZN stain has resulted. Epitope mapping of this recombinant HSP70 is necessary to find specific monoclonal antibodies for diagnosis of MAP in different animal tissues.

## Conclusion

Anti-HSP70 produced in this study offers an opportunity for improved diagnosis of MAP in animal tissues. It could also be used in screening of cases especially in studies focusing on abattoir surveys of MAP infection particularly if automated IHC protocols are used and in studies on the pathogenesis of MAP.

## Additional file

Additional file 1:
**Table S1.** Information regarding the samples included in the study. (DOCX 17 kb)

## Abbreviations

DAB: 3, 3 – Diaminobenzidine
DTT: Dithiothreitol
HSP70: Heat shock protein 70 Kd
IHC: Immunohistochemistry
MAP: *Mycobacterium avium subspecies paratuberculosis*
PCR: Polymerase chain reaction
RT: Room temperature
SUMO: Small ubiquitin like modifier
TGED: A buffer containing, Tris HCl, Glycerol, EDTA and Dithiothreitol
