# Supplementary material for: Application of antibodies to recombinant heat shock protein 70 in immunohistochemical diagnosis of mycobacterium avium subspecies paratuberculosis in tissues of naturally infected cattle
Source: Ir Vet J. 2017 Mar 24;70:10. doi: 10.1186/s13620-017-0088-7 (PMC5364614; doi:10.1186/s13620-017-0088-7)
Supplement: Additional file 1: — Table S1. Information regarding the samples included in the study. (DOCX 17 kb) [file 13620_2017_88_MOESM1_ESM.docx]

Additional file 1. Table S1: Information regarding the samples included in the study

| **ID** | **Gross lesion** | **Microscopic classification** | | **ZN** | **IHC** | **Tuberculoid** | **Lepramatous** | **Culture** | **IS 900**  **PCR** |
| --- | --- | --- | --- | --- | --- | --- | --- | --- | --- |
|  |  | **Buergelt** | **Perez** |  |  |  |  |  |  |
| 07JD06 | None | Moderate | Multifocal | ₊ | ₊₊₊ | yes |  | + | + |
| 07JD09 | None | Mild | Focal | ₊ | ₊₊₊ | yes |  | + | + |
| 07JD18 | Reddening of the ileocaecal valve | Moderate | Multifocal | ₊ | ₊₊₊ | yes |  | - | NA* |
| 07JD44 | None | Mild | Focal | ₊₊ | ₊₊ | Intermediate |  | + | + |
| 07JD53 | None | Mild | Focal | ₊₊ | ₊₊₊ |  | yes | + | + |
| 07JD70 | None | Mild | Focal | ₊ | ₊₊₊ | yes |  | - | NA |
| 07JD340 | Reddening of the ileocaecal valve | Mild | Focal | ₊ | ₊₊₊ | yes |  | - | NA |
| 07JD377 | Slight reddening of ileocaecal valve | Moderate | Multifocal | ₊ | ₊₊₊ | yes |  | + | + |
| 07JD574 | Corrugation of ileocaecal mucosa | marked | Diffuse | ₊₊₊ | ₊₊₊ |  | yes | - | NA |
| 07JD575 | Chronic congestion, corrugation, oesophastomum nodules | Moderate | Multifocal | ₊₊₊ | ₊₊₊ |  | yes | + | + |
| 07JD577 | Corrugation of ileocaecal mucosa, parasite nodule | Mild | Focal | ₊₊ | ₊₊ | Intermediate |  | - | NA |
| 07JD587 | Reddening of the ileocaecal valve | Mild | Focal | ₊ | ₊₊ | yes |  | - | NA |
| 07JD593 | Reddening of the ileocaecal valve | marked | Diffuse | ₊ | ₊₊₊ |  | yes | - | NA |
| 07JD690 | Corrugation of ileocaecal mucosa, parasite nodule | Mild | Focal | 0 | ₊₊₊ | yes |  | - | NA |
| 07JD859 | Congestion and corrugation, some nodules on mucosa | Mild | Focal | ₊ | ₊₊₊ | yes |  | - | NA |
| 07JD862 | Reddening of the ileocaecal valve | marked | Diffuse | ₊₊₊ | ₊₊₊ |  | yes | - | NA |
| 07JD873 | Reddening of the ileocaecal valve | Moderate | Multifocal | ₊₊ | ₊₊ | Intermediate |  | - | NA |
| 07JD888 | None | Mild | Focal | ₊ | ₊₊ | yes |  | - | NA |
| 07JD962 | None | Mild | Focal | ₊ | ₊₊ | yes |  | - | NA |
| 07JD967 | None | Mild | Focal | 0 | ₊₊ | yes |  | - | NA |
| 07JD971 | None | Moderate | Diffuse | ₊₊ | ₊₊₊ | Intermediate |  | - | NA |
| 07JD993 | Reddening of the ileocaecal valve | Moderate | Multifocal | ₊₊ | ₊₊₊ | Intermediate |  | - | NA |
| 07JD997 | Reddening of the ileocaecal valve | Mild | Multifocal | ₊₊₊ | ₊₊₊ |  | yes | - | NA |
| 07JD1041 | None | Mild | Focal | ₊₊ | ₊₊ | Intermediate |  | - | NA |
| 07JD1084 | Reddening of the ileocaecal valve | Mild | Focal | ₊₊ | ₊₊₊ | Intermediate |  | - | NA |
| JP1 | Systemic disease with corrugation of entire length of intestine | Severe | Diffuse | +++ | +++ |  | Yes | + | = |
| GBTBC 1 | Not provided | Severe | Multifocal - diffuse | +++ | +++ |  | Yes | Unknown** |  |
| GBTBC2 | Not provided | severe | Multifocal - diffuse | +++ | +++ |  | Yes | Unknown | Unknown |
| GBTBC3 | Not provided | severe | Multifocal - diffuse | +++ | +++ |  | Yes | Unknown | Unknown |
| GBTBC4 | Not provided | Severe | Multifocal-diffuse | +++ | +++ |  | Yes | Unknown | Unknown |
| GST 22 | Not provided | Severe | Multifocal to diffuse | +++ | +++ |  | Yes | Unknown | Unknown |
| GST 26 | Not provided | Severe | Multifocal to diffue | +++ | +++ |  | Yes | Unknown | Unknown |
| GST9 | Not provided | severe | Multifocal to diffuse | +++ | +++ |  | Yes | Unknown | Unknown |
| GST 23 | Not provided | severe | Multifocal to diffuse | +++ | +++ |  | Yes | Unknown | Unknown |
| GST2 | Not provided | Severe | Multifocal to diffuse | +++ | +++ |  | Yes | Unknown | Unknown |

NA* : The bacteria was not isolated, so PCR was performed.

Unknown: There was no information if the cases had been confirmed by either PCR or culture.
